# Exploration of the core metabolism of symbiotic bacteria

**DOI:** 10.1186/1471-2164-13-438

**Published:** 2012-08-31

**Authors:** Cecilia Coimbra Klein, Ludovic Cottret, Janice Kielbassa, Hubert Charles, Christian Gautier, Ana Tereza Ribeiro de Vasconcelos, Vincent Lacroix, Marie-France Sagot

**Affiliations:** 1 BAMBOO Team, INRIA Grenoble-Rhône-Alpes, Villeurbanne, France; 2 Laboratoire de Biométrie et Biologie Évolutive, Université de Lyon, Université Lyon 1, CNRS, Villeurbanne, UMR5558, France; 3, UMR1089 Xénobiotiques INRA-ENVT; 4, UMR203 Biologie Fonctionnelle Insectes et Interactions (BF2I), INRA, INSA-Lyon, Villeurbanne, France; 5, Laboratório Nacional de Computação Científica (LNCC), Petrópolis, Brazil

## Abstract

**Background:**

A large number of genome-scale metabolic networks is now available for many organisms, mostly bacteria. Previous works on minimal gene sets, when analysing host-dependent bacteria, found small common sets of metabolic genes. When such analyses are restricted to bacteria with similar lifestyles, larger portions of metabolism are expected to be shared and their composition is worth investigating. Here we report a comparative analysis of the small molecule metabolism of symbiotic bacteria, exploring common and variable portions as well as the contribution of different lifestyle groups to the reduction of a common set of metabolic capabilities.

**Results:**

We found no reaction shared by all the bacteria analysed. Disregarding those with the smallest genomes, we still do not find a reaction core, however we did find a core of biochemical capabilities. While obligate intracellular symbionts have no core of reactions within their group, extracellular and cell-associated symbionts do have a small core composed of disconnected fragments. In agreement with previous findings in *Escherichia coli*, their cores are enriched in biosynthetic processes whereas the variable metabolisms have similar ratios of biosynthetic and degradation reactions. Conversely, the variable metabolism of obligate intracellular symbionts is enriched in anabolism.

**Conclusion:**

Even when removing the symbionts with the most reduced genomes, there is no core of reactions common to the analysed symbiotic bacteria. The main reason is the very high specialisation of obligate intracellular symbionts, however, host-dependence alone is not an explanation for such absence. The composition of the metabolism of cell-associated and extracellular bacteria shows that while they have similar needs in terms of the building blocks of their cells, they have to adapt to very distinct environments. On the other hand, in obligate intracellular bacteria, catabolism has largely disappeared, whereas synthetic routes appear to have been selected for depending on the nature of the symbiosis. As more genomes are added, we expect, based on our simulations, that the core of cell-associated and extracellular bacteria continues to diminish, converging to approximately 60 reactions.

## Background

We now have at our disposal the full metabolic network based on genomic data for hundreds of species, mostly bacteria. The level of annotation is however widely heterogeneous across species, making it crucial for any comparative analysis to carefully choose a set of species for which we can guarantee a good enough annotation, and a same procedure for inferring the metabolic network from the annotated genomes.

One question commonly raised by the availability of many complete genome sequences is the number and content of a minimal set of protein-coding genes necessary to sustain a living cell [1-3], which has been investigated using experimental and computational approaches [4-15]. One such method identifies essential genes based on those shared among genomes in a comparative analysis of diverse taxa [1,3,7,8,14]. Some studies included obligate host-dependent bacteria as a possibility for defining minimal gene sets in more specific and naturally occurring conditions [7,8]. The minimal gene sets proposed were not enriched in metabolic genes [3,7,8,12,14] and the corresponding pathways often presented missing steps [8,16]. These gaps may be due to non-orthologous gene displacement (NOGD) (*i.e.*, the presence of non-orthologous, paralogous or unrelated, genes for the same function in different organisms) [16] whose encoded enzymes have been defined as analogous (as opposed to homologous) and may be structurally unrelated [17]. In comparative analyses of reaction sets instead of genes, NOGD has a reduced impact because different orthologous families encoding a single enzymatic capability are often represented by a same reaction. Another possible explanation for incomplete pathways is the use of different alternative routes, which recently have been defined as alternologs (*i.e.*, branches that proceed via different metabolites and converge to the same end product) [18]. Their origin is closely related to different environmental metabolite sources and lifestyles among species [18]. Since metabolism is a core function expected to be required for sustaining life [19], and the core size may continue decreasing as more genome sequences appear [9,20], alternative approaches relaxing the requirement for ubiquity were proposed for analysing either prokaryotes [9,13,21,22] or species from the three domains of life [20,23,24]. One such example is the search for proteins commonly present (persistent) instead of strictly conserved everywhere [20]. On the other hand, conserved portions of metabolism are found in lifestyle groups of bacteria [3].

Small-scale comparative analyses of a selection of metabolic pathways were performed investigating each one individually [25,26] or grouped in one functional module [27]. On the other hand, larger-scale comparative analysis were carried out in other papers but the question put in each case was different, related either to the proportion of metabolic genes in an organism, in absolute [28] or classified according to lifestyle [29,30], or related to the association between ecological strategies and growth rate [31]. The notion of a core metabolism, meaning common elements, has been previously studied. However, this was done by comparing all known strains of a same species, namely, *Escherichia coli*[32]. This approach of analysing metabolism as a single network allows a global view of functional processes, which was enabled by metabolic reconstruction methods based on genomic data [33-37].

Here, we work at the level of whole metabolic networks for each organism and we analyse the core small molecule metabolism (*i.e.*, its conserved portion) of different lifestyle bacteria, aiming to characterise the contribution of each lifestyle group in the reduction of the common set of metabolic capabilities shared by the whole dataset. As concerns the impact of the obligate intracellular group, the question could be reformulated as the reactions which could not be dispensed and/or outsourced to the host in the course of genome compaction. Our major goals were to have a representative diversity in the symbiotic associations, a balanced amount of organisms in each lifestyle group, and as few biases as possible that might be related to the use of different annotation pipelines which is important when performing comparative analyses. We address this by comparing the presence of metabolic reactions as well as biochemical capabilities based on a partial Enzyme Commission (EC) number analysis at level 3 (*e.g.*, 2.5.1.-) [38]. The purpose of the first is to be stringent although partially dealing with NOGD (see Additional file 1 for an example), while the purpose of the latter is to be more relaxed and to compare common functional capabilities in a broader sense. There are two possible advantages to this. One is to deal with enzymatic activities for which it was not possible to assign a full EC number during the functional annotation of a genome which resulted in partial EC numbers that do not denote a specific reaction. The second reason is to try to address the issue of alternologs, *e.g.*, alternative amino acid biosynthetic pathways that are often composed of enzymes which have the same partial EC numbers at level 3 (in the two alternative phenylalanine biosynthetic pathways, the partial EC numbers are ec:5.4.99, ec:4.2.1 and ec:2.6.1). We also analysed the metabolites that each bacterium potentially acquires from its environment in order to relate them to the set of common metabolic functions found for each lifestyle group.

## Methods

### Dataset

**Figure 1:**
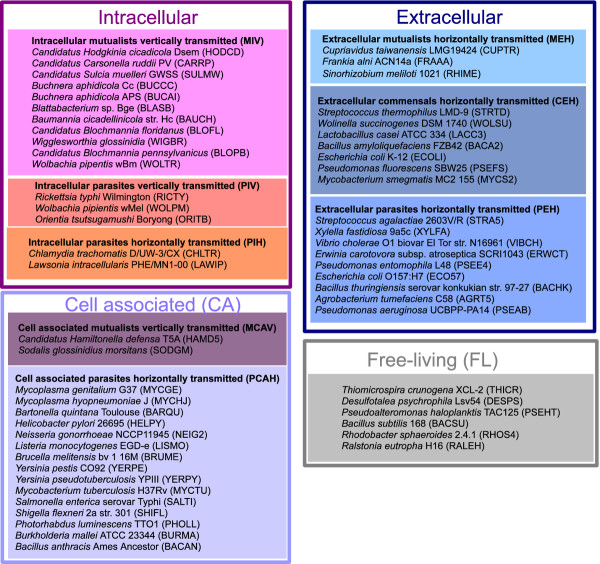
**The lifestyle dataset consists of 58 bacteria.** The 58 bacteria were classified in 4 broader lifestyle groups based on the location of the bacterium in its host: *obligate intracellular* INTRA, *cell associated* CA, *extracellular* EXTRA (16, 17 and 19 organisms, respectively) and the control group FL. We further grouped them in subcategories on the basis of the association type (*mutualism* M, *commensalism* C, *parasitism* P) and the transmission mode (*vertical* V, *horizontal* H). INTRA includes only obligate intracellular bacteria, whereas CA includes bacteria which are facultatively intracellular, live on the surface of the host cell or are extracellular with a described intracellular step. These two groups have bacteria with obligate associations with their host (the totality of the individuals of the first group and 76% of the second one). EXTRA presents only bacteria that are facultatively associated with their host and are also free-living. The group FL has been used as a control group for all the analyses performed and includes organisms that have none of the three types of associations used for the grouping in the HAMAP information on interactions [40]. For that reason we included in FL one representative of each taxonomic class present in the dataset depending on the availability in the Microscope platform [39]. Two *Mycoplasma* species were classified in the CA group as they live on the surface of the host cell, although in another study they have been grouped together with the INTRA because of their reduced genome and the invariant environment within the hosts [44]. The codes for the organisms are the ones from HAMAP [40].

We selected 58 bacteria from the MicroScope platform [39] and we carefully classified them according to their lifestyle based on the HAMAP information on interactions [40] and the information provided in the literature. The broader lifestyle groups take into account the location of the bacterium in its host, constituting four groups: *obligate intracellular* INTRA, *cell associated* CA, *extracellular* EXTRA (16, 17 and 19 organisms, respectively) and the control group *free-living* FL. We further grouped them in subcategories on the basis of the association type and transmission mode. The lifestyle groups and the abbreviations are given in Figure 1. The full list of bacteria selected and their detailed classification is given in Additional file 2. The data on genes, metabolites and reactions were obtained from MicroCyc/MicroScope [39]. MicroCyc is a collection of microbial Pathway/Genome Databases which were generated using the PathoLogic module from the Pathway tools software [41] which computes an initial set of pathways by comparing a genome annotation to the metabolic reference database MetaCyc [42]. Using these databases as input, the metabolic networks of the 58 bacteria were obtained from MetExplore [43]. It is important to notice that the completeness of metabolic network reconstructions is a current limitation as some reactions remain to be discovered and will be missing in the model while some false positive reactions may be wrongly included in the network. On the other hand, reactions shared by most bacteria are less likely to be missing in current datasets than organism-specific reactions, favouring the kind of analyses performed in the present work. The data on metabolic pathways were obtained from MetaCyc [42].

### Core metabolism and core enzymatic function

Our analysis is restricted to the small molecule metabolism as defined in the MetaCyc/BioCyc databases [42,45], *i.e.* small molecule reactions are those in which all participants are small molecules, hence reactions involving one or more macromolecules such as proteins or nucleic acids are not represented. The comparisons of compound and reaction sets are based on the BioCyc labels [45], *e.g.*, the last reaction of glycolysis consists in the transformation of phosphoenolpyruvate, ADP and H^ + ^into pyruvate and water, and its label is PEPDEPHOS-RXN. The compounds found in the metabolic networks are those which are involved as substrates or products in the inferred reactions. All metabolites directly provided by the environment and not involved in any reaction as substrate are not included.

The presence of a metabolic core, *i.e.*, of a conserved set of elements in bacteria with different lifestyles, was analysed in terms of common compounds, common reactions and common partial EC number sets. The core metabolism was obtained by computing the intersection of the sets of reactions (resp. compounds and partial EC numbers) for each species. The panmetabolism was obtained by computing the union of these sets. The variable metabolism is the difference between pan- and core metabolism, *i.e.*, the set of elements that are missing from at least one bacterium. These definitions were introduced by Vieira *et al.*[32], however they worked with strains of a same species whereas here we compare different species.

The metabolic networks of the 58 bacteria were obtained from MetExplore [43]. The macromolecules, as defined by BioCyc, were filtered out for all the analyses. The analyses were performed using R [46], as were the graphics. The igraph package [47] was adopted for analysing graphs.

For the analysis of the core enzymatic functions we used the EC number classification [38] which consists in a specific numerical identifier (*e.g.* 2.5.1.3) based on the chemical reactions a given enzyme catalyses. We worked with partial EC number sets at level 3 (*e.g.* 2.5.1.-), leaving the fourth digit open. The first digit represents which of the six main classes the enzyme belongs to (*e.g.* 1 for oxidoreductases; 2 for transferases). The following 3 digits provide a more detailed description of the enzymatic activity.

### Connectivity in the reaction graph

We analysed the connectivity of the core metabolic network to check if the common reactions would be connected among themselves, *i.e.*, the produced metabolites would be consumed by other reactions in a chain of biochemical transformations. For that, the metabolic networks of the dataset were modelled as reaction graphs. In such a graph, nodes represent reactions, and arcs (*i.e.*, directed edges) between two reactions represent a compound which is produced by one reaction and consumed by the other. We set filters to exclude pairs of cofactors (*i.e.*, ADP+P_*i*_→ ATP, NAD^+^+H^+ ^→ NADH; for the full list see the MetExplore documentation) and current compounds (*e.g.*, water, proton, *CO*_2_, phosphate, diphosphate, *NH*_3_, *H*_2_*O*_2_ and *O*_2_), which otherwise would connect unrelated reactions [48].

Since we were working with the common reactions of a group of organisms, we computed the union graph of all the metabolic networks modelled as reaction graphs. We then calculated the graph induced by the common set of reactions, *i.e.*, the subgraph containing the nodes corresponding to these reactions as well as the arcs that link them. After that, we checked for the presence of connected components, *i.e.*, whether for every pair of nodes there is an undirected path.

In the case of the common partial EC number sets, we checked whether the reactions corresponding to each one of the partial EC numbers, *i.e.*, one reaction for each partial EC number, are connected in the metabolic networks. We analysed this in the union of all metabolic networks of the dataset (or of lifestyle groups) modelled as a reaction graph, as well as in the graph of each organism. This analysis was performed using MOTUS [49].

### Controlling for the impact of small networks

We controlled for the impact of bacteria with very reduced genomes on the size of the common set of reactions (resp. partial EC number sets). The six organisms which possess the smallest reaction sets (resp. partial EC number sets) were successively removed (*i.e.*, by forming subgroups from 57 to 53 bacteria) and the intersections of the remaining subgroups were recomputed. These six organisms are: “*Candidatus* Hodgkinia cicadicola” (HODCD), “*Candidatus* Carsonella ruddii” (CARRP), “*Candidatus* Sulcia mueller” GWSS (SULMW), *M. genitalium* (MYCGE), *Buchnera aphidicola* Cc (BUCCC) and *Mycoplasma hyopneumoniae* (MYCHJ). All possible orders for removing them were tested, and then the mean of the intersection sizes for each subset size of organisms was calculated. We also performed the same analysis by removing the eight bacteria with the smallest sets of reactions (resp. partial EC numbers).

### Decay of the common reactions in the different lifestyle groups

Next, we checked whether there were reactions common to subsets of organisms within the same lifestyle group. To do so, for each lifestyle group *l* (*l*=*INTRA, CA, EXTRA*) having *n*_*l*_ organisms, we randomly drew *x* (2≤*x*≤*n*_*l*_−1) organisms and computed the intersection (*y*) of their reaction sets. This was repeated 1000 times. In order to test if, when adding more species, the size of the intersection of reaction sets was expected to decrease to zero, we fitted exponential (*E*_*l*_) and logistic (*L*_*l*_) models to the data obtained for each lifestyle group *l*. Assuming normally distributed residuals, ϵ∼N(0,σ), these models are given by:

(1) $$\begin{array}{llll} E_{l}\;\;: & {\bar{y}}_{l}=N_{l}*\exp(-r_{l}*x_{l})+\alpha_{l}+\epsilon_{l} & & \end{array}$$

(2) $$\begin{array}{llll} L_{l}\;\;: & {\bar{y}}_{l}=\frac{r_{l}}{(\frac{r_{l}}{N_{l}}-\frac{r_{l}}{\alpha_{l}})*\exp(-r_{l}*x_{l})+\frac{r_{l}}{\alpha_{l}}}+\epsilon_{l} & & \end{array}$$

where $\bar{y}$ represents the mean of the intersection of the reaction set over the 1000 simulations, *x* is the subset size (*i.e.*, the number of organisms drawn), *α*_*l*_ is the asymptote, *r*_*l*_ is the decay rate and *ϵ*_*l*_ is the residual of the *l*^*th*^lifestyle group. *N*_*l*_is theoretically defined as the mean of the reaction sets for an empty subset size ( $\bar{y}$_*l*_ for *x*_*l*_=0). A null intersection of the reaction sets corresponds to an asymptote *α*=0.

Preliminary analyses showed the strong impact of the two *Mycoplasma* species on the intersection size due to their reduced genomes (data not shown). Thus, both species were removed from the CA group for this simulation. We used the R package nlstools [50] for model parameter estimation.

### Differential random loss of enzymes

In order to rule out the possibility that the small intersection of partial EC number sets could be simply explained by a differential random loss of enzymes during genome reduction of the intracellular symbionts, we simulated the MIV (Mutualistic Intracellular Vertically transmitted, see Figure 1 for group names) partial EC number sets starting from bacteria of the EXTRA group. This was restricted to the *Gammaproteobacteria* of both groups. To do so, for each *Gammaproteobacteria* of the MIV group (7 organisms), we randomly picked a corresponding EXTRA *Gammaproteobacterium* and we randomly removed reactions from its set of reactions, until we reached the size of the corresponding MIV metabolic network. Then, we replaced each remaining reaction by its partial EC number at level 3, and removed redundant partial EC numbers from this set. We therefore obtained a group of simulated MIV networks for which we computed the union, intersection and average size of their partial EC number sets. This whole procedure was repeated 1000 times. Additionally, we aimed to test the differential random loss of biochemical capabilities, meaning the loss of partial EC numbers (at level 3). For that, we performed a similar procedure to the one explained above, however we stopped removing reactions when we reached the size of the MIV partial EC number set. We used a Monte-Carlo test from the R ade4 package [51] to compare simulated and observed values.

### Metabolites potentially acquired from the environment

In order to identify which metabolites each bacterium potentially acquires from its environment (*i.e.*, potential inputs), we used the Borenstein method [52]. For this, the metabolic network of each bacterium was modelled as a directed compound graph, whose nodes are metabolites and arcs link a substrate to a product of a reaction. The cofactors and current compounds were filtered. We implemented a version of the Borenstein method using the igraph package [47]. In order to cope with possible common inputs missed by the metabolic network reconstruction, we allowed distance one from the topological precursors if they were already assigned as input in another bacterium, and we grouped and compared them among organisms. In this analysis, the following compounds were removed since they are only produced by reactions which also involve macromolecules: dADP, dCDP, dUDP, dGDP. Hence, a systematic search for the inputs in the small molecule metabolism would indicate these compounds as potential inputs, whereas they in fact can be produced by the cell.

## Results

### Data overview

**Figure 2:**
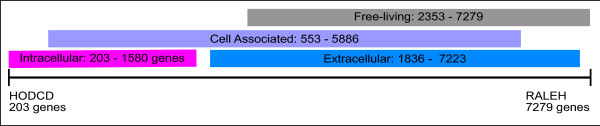
**Range of the total number of genes in the different lifestyle groups.** Abbreviations of group names are those from Figure 1.

The total number of genes varies greatly among the 58 bacteria, ranging from 203 genes for “*Ca.* Hodgkinia cicadicola” (HODCD) to 7279 genes for *Ralstonia eutropha* (RALEH) (Figure 2 and Additional file 3). We noticed that the number of genes is greater in the EXTRA than in the INTRA, in agreement with the reduced genomes related to the intracellular lifestyle [53,54].

The three most frequent taxonomic classes among all the organisms analysed are *Gammaproteobacteria*, *Alphaproteobacteria* and *Bacilli*. These classes are well distributed in relation to the number of genes (Additional file 3), with no correlation observed between the two factors (Kruskal-Wallis test, *p*=0.65).

**Figure 3:**
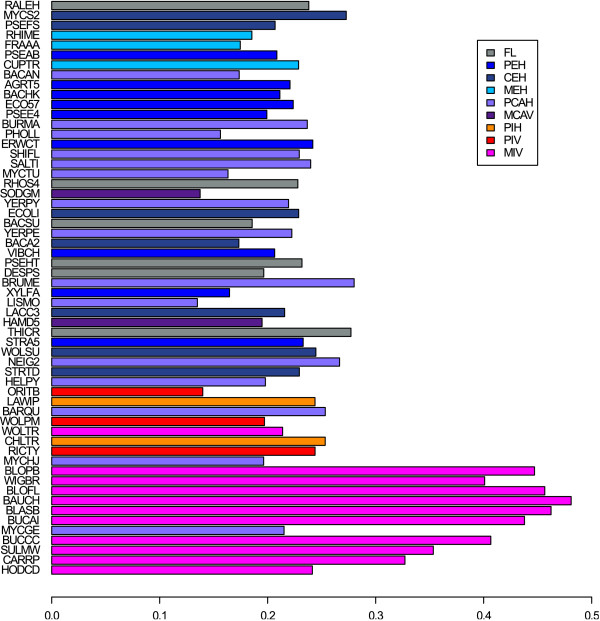
**Ratio of the number of metabolic genes over the total number of genes.** The colours correspond to the lifestyle groups (see Figure 1 for the abbreviations of group names) and bacteria are ordered by total number of genes.

The number of metabolic genes ranges from 49 for “*Ca.* Hodgkinia cicadicola” (HODCD) to 1970 for *Mycobacterium smegmatis* (MYCS2). As for the total number of genes, the number of metabolic genes is greater in the EXTRA as compared to the INTRA bacteria. However, the ratio of the number of metabolic genes over the total number of genes shows important differences depending on the organism (Figure 3). For the organisms in the groups other than MIV, the mean ratio of metabolic genes is 0.21 ± 0.08. By contrast, the mean ratio of metabolic genes for the MIV bacteria is 0.38 ± 0.18 and even reaches 0.48 for “*Candidatus* Baumannia cicadellinicola” (BAUCH), being significantly different from the bacteria of the other groups (Wilcoxon test, *p*<0.001).

**Figure 4:**
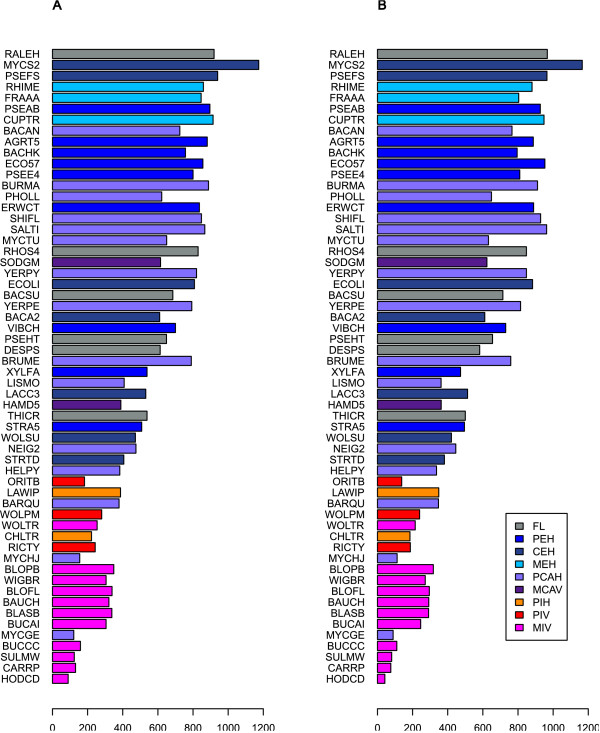
**Total number of elements in the metabolic network of each bacterium in the dataset.** (**A**) Total number of compounds. (**B**) Total number of reactions. The colours correspond to the lifestyle groups (see Figure 1 for the abbreviations of group names). Bacteria are ordered by total number of genes.

Among the 58 bacteria analysed, the number of compounds and reactions follows almost the same trend as the number of metabolic genes, from 98 compounds and 42 reactions for “*Ca.* Hodgkinia cicadicola” (HODCD) to 1381 compounds and 1166 reactions for *M. smegmatis* (MYCS2) (Figure 4).

### Core metabolism in the whole set of bacteria

#### Shared compounds and reactions

**Figure 5:**
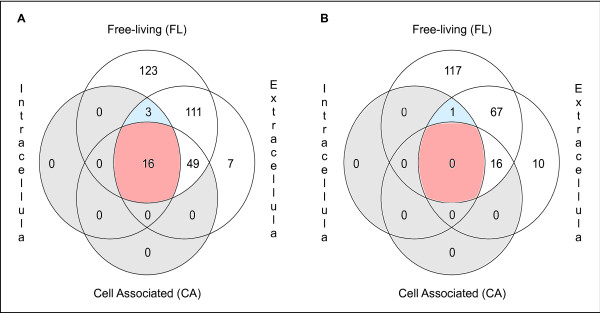
**Size of the intersection of compound (A) and reaction (B) sets among groups of lifestyle. Red**: number of compounds/reactions common to the whole dataset. **Blue**: number of compounds/reactions common the intracellular, extracellular and FL groups.

For the whole set of organisms, there are only 16 common compounds (Additional file 4) which correspond to amino acids, cofactors, ions and metabolites involved in the synthesis of nucleic acids. No small-molecule metabolic reaction is common to every organism of the dataset (Figure 5 and Additional file 5). The 16 compounds shared by the 58 organisms are therefore not involved in the same reactions in each organism. The full list of reactions analysed with the number of bacteria that possess them and the list of the organisms that lack them is presented in the Additional file 6.

#### Core enzymatic function based on an EC number analysis

**Figure 6:**
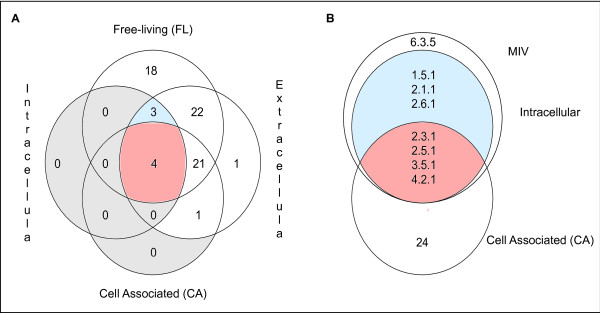
**Size of the intersection of partial EC number sets.** (**A**) Intersection of the partial EC number sets among groups of lifestyle. **Red**: number of compounds/reactions common to the whole dataset. **Blue**: number of compounds/reactions common to the intracellular, extracellular and FL groups. (**B**) Detailed intersection of the partial EC number sets of the MIV, intracellular and CA groups. **Red**: the 4 partial EC numbers common to the whole dataset. **Blue**: 3 partial EC numbers present in the intracellular, extracellular and FL groups and absent in the CA group.

We found only four partial EC numbers common to all 58 bacteria: two transferases (2.3.1 and 2.5.1), one hydrolase (3.5.1) and one lyase (4.2.1) (Table 1, Figure 6A and Additional file 7). They correspond to 235 reactions in the union of all the reactions of our dataset. We searched for any four reactions, each corresponding to one of the four partial EC numbers, that are connected in the metabolic networks. In the union of all metabolic networks of the dataset, the graph induced by these 235 reactions has 418 arcs and forms 20 connected components apart from 84 isolated reactions. There are 30 occurrences of the four reactions (one for each of the four partial EC numbers) connected in the union of the metabolic networks. In the reaction graph of each organism, we found this connected pattern of four reactions in the graphs of 28 organisms. The common set of partial EC numbers in the whole dataset thus corresponds to a connected portion of the metabolic network of 28 bacteria out of the 58 analysed. This subset of bacteria represents most lifestyle groups described in this work, ranging from obligate intracellular to free-living as well as from mutualists to parasites.

**Table 1:** Partial EC numbers common to the whole dataset

**EC number**	**Classification**
**2**	**Transferases**
2.3	Acyltransferases
2.3.1	Transferring groups other than aminoacyl groups
2.5	Transferring alkyl or aryl groups, other than methyl groups
2.5.1	Transferring alkyl or aryl groups, other than methyl groups
	(only subclass identified to date)
**3**	**Hydrolases**
3.5	Acting on carbon-nitrogen bonds, other than peptide bonds
3.5.1	In linear amides
**4**	**Lyases**
4.2	Carbon-oxygen lyases
4.2.1	Hydro-lyases

The detailed description of the 4 partial EC numbers shared by the whole dataset includes 3 classes of enzymes: transferase, hydrolase and lyase.

#### Controlling for the impact of small networks

Clearly, we can expect that the inclusion of bacteria with very reduced genomes will have a large impact on the size of the intersection. However, it remains unclear if the small size of the intersection could be explained only by this. We found that the shared reaction sets, obtained when decreasing the number of organisms, range from zero to five and the mean varies from 0.2 to 2.5. There are 12 common reactions without the six bacteria with the smallest reaction sets, 7 take part in the biosynthesis of peptidoglycan, which is a cell wall precursor. These reactions do not complete this biosynthetic pathway (there are 4 missing steps, two present in 51 organisms and the other two in less than half of the dataset). Among the other reactions, there is an inorganic pyrophosphatase, a reaction involved in folate transformations and a couple of reactions which take part in purine nucleotides *de novo* biosynthesis. Removing the eight bacteria with the smallest reaction sets resulted in similar intersection sizes ranging from zero to 13 reactions and mean varying from zero to 5.4. Hence, the intersection sizes did not increase much without the bacteria with reduced metabolism.

On the other hand, these bacteria had a greater impact on the common partial EC number sets. The size of the intersections when decreasing the number of organisms ranges from 4 to 19 and the mean varies from 5 to 16. This size increases to 23 partial EC numbers without the same six bacteria. When removing the eight bacteria with the smallest sets, the sizes of the intersections increase, ranging from 4 to 27, reaching 30 partial EC numbers. Therefore, a core of biochemical capabilities composed of 30 partial EC numbers is present in 50 bacteria out of the 58 analysed. This set is made of all classes of enzymes: 3 oxidoreductases, 13 transferases, 4 hydrolases, 3 lyases, 5 isomerases and 2 ligases, which correspond to 958 reactions in our dataset (Table 2).

**Table 2:** Partial EC number set common to 50 bacteria of the dataset

**Classes**	**Partial EC**	**N^*o*^ reactions**
	1.1.1	134
Oxidoreductases	1.2.1	50
	1.5.1	15
	2.1.1	46
	2.1.2	6
	2.2.1	8
	2.3.1	47
	2.4.1	69
	2.5.1	51
Transferases	2.6.1	51
	2.7.1	71
	2.7.2	8
	2.7.4	19
	2.7.6	4
	2.7.7	23
	2.7.8	15
Hydrolases	3.1.3	39
	3.5.1	32
	3.5.4	15
	3.6.1	26
	4.1.1	49
Lyases	4.1.2	22
	4.2.1	62
	5.1.1	10
	5.1.3	16
Isomerases	5.3.1	21
	5.4.2	9
	5.4.99	10
Ligases	6.3.2	19
	6.3.4	11
Total	30	958

Partial EC number set common to 50 bacteria out of the 58 present in our dataset and the number of reactions in the dataset corresponding to each partial EC number. The 8 bacteria which possess the smallest partial EC number sets were removed to calculate the common set.

### Core metabolism according to lifestyle groups

#### Shared compounds and reactions

The same analyses performed for the whole dataset were also applied to the different lifestyle groups (Figure 5 and 6). The aim was to describe the influence of these groups on the size and composition of the common sets of compounds, reactions and partial EC numbers as well as on the union of each of these sets of elements. As a first overview of the two opposing groups in terms of the size of the metabolic networks, *i.e.*, the INTRA and the EXTRA groups, we notice that the size of the union of the compound sets (*i.e.*, the pan-metabolome) is quite large when compared to the mean number of compounds, indicating a relative diversity of the metabolome in these organisms (Table 3). By contrast, the sizes of the intersections of compound and reaction sets for the same groups are considerably different. Therefore, the universe of compounds and reactions of the intracellular bacteria is quite diverse, while common elements are far less abundant.

**Table 3:** Compound and reaction sets among lifestyle groups

	**Compounds**	**Reactions**		
	**Mean / Union**	**Intersection / Mean**	**Mean / Union**	**Intersection / Mean**
Intracellular	39%	8%	30%	0.5%
Cell Associated (CA)	39%	11%	34%	3%
Extracellular	41%	25%	36%	12%
Free-living (FL)	52%	43%	46%	28%

Ratio of the mean size over the union size, as well as the ratio of the intersection size over the mean size, of the compound and the reaction sets among the different lifestyle groups of bacteria.

#### Core metabolism in the extracellular bacteria

We found 186 compounds and 94 reactions shared by the 19 extracellular bacteria. The compounds include nucleosides, amino acids, carbohydrates, cofactors, while the reactions are involved in metabolic pathways, such as glycolysis, nucleotide and amino acid biosynthesis and degradation pathways, and peptidoglycan biosynthesis (Additional file 8). Most of them (88%) are classified as biosynthetic processes according to the metabolic processes defined in the BioCyc databases (Additional file 9). These reactions shared by the EXTRA are not connected in the reaction graph induced by these 94 reactions, which is composed of 10 connected components apart from 17 isolated reactions. The largest component has 26 reactions which are involved in pyrimidine ribonucleotides *de novo* biosynthesis, peptidoglycan and amino acid biosynthesis.

#### Core metabolism in the cell associated bacteria

The CA bacteria showed a considerable reduction in the common elements which are 67 compounds and 17 reactions. Even with this reduction, similar categories of compounds as for the EXTRA were found, whereas the reactions observed take part in fewer metabolic pathways: glycolysis and nucleotide biosynthesis and degradation pathways. Most of them (82%) are classified as biosynthetic processes (Additional file 9). This group is supposed to be intermediate between the INTRA and the EXTRA ones, thus presenting a broad diversity of genome sizes. In this group, the two bacteria with smallest genomes are *M. genitalium* (MYCGE) and *M. hyopneumoniae* J (MYCHJ) which are obligate parasites that have undergone extreme reductive genome evolution [10,55,56]. This pair of organisms is the one that most influences the small intersection of the CA group. Hence, the intersection of the elements of the CA bacteria without the two *Mycoplasma* species increases to 167 compounds and 88 reactions. These values are similar to the ones found for the EXTRA, the reactions take part in the same metabolic pathways observed for this group and the classification into biosynthetic and degradation processes present similar ratios (Additional file 9).

#### Core metabolism in the obligate intracellular bacteria

The INTRA share 19 compounds and one reaction (3.5.1.88-RXN, MetaCyc [42]). Indeed, the MIV is the group mainly responsible for this reduction. The common compounds still include the same ones mentioned for the EXTRA group. The only shared reaction is not assigned to participate in any metabolic pathway in MetaCyc.

As there is only one reaction common to INTRA, it is not possible to analyse whether there is a majority of biosynthesis reactions in their core as we found in the EXTRA and CA. Instead, we analyse the content of biosynthesis and degradation reactions in the variable metabolism (see Methods for definition). The total number of reactions in the variable metabolism is 704 (62% in biosynthetic and 24% in degradation processes) for the intracellular group while it is 2049 (38% in biosynthesis and 35% in degradation) in the EXTRA (Additional file 9). The variable metabolism of intracellular bacteria is therefore enriched in biosynthetic reactions (Fisher exact test, *p*<10^−15^) and depleted in degradation reactions (Fisher exact test, *p*<10^−8^).

#### Controlling for the structuring of MIV

As mentioned, the absence of a metabolic core common to all symbionts is mainly caused by the absence of such a core within the MIV group. We further analysed this group and we found two subgroups with opposite patterns of reaction presence/absence (Additional file 1 for detailed results and methods), which can be directly related to the role of the symbiont in the mutualistic relationship [25,57-64]. Intersecting the reaction sets of each subgroup with non MIV organisms did not increase much the common sets, thus the structuring of the MIV symbionts does not explain the reduced number of reactions shared by the bacteria analysed.

#### Decay of the common reactions in the different lifestyle groups

**Figure 7:**
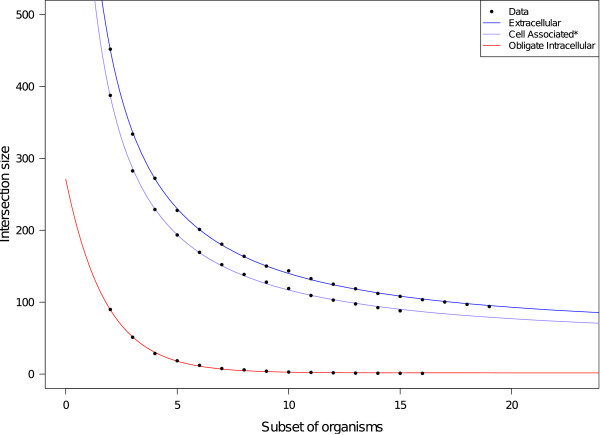
**Decay of the common reactions in the different lifestyle groups.** Data is the mean value of the 1000 simulations of the intersection of reaction sets for the different subsets of organisms for each lifestyle group. The red curve corresponds to the fitted exponential model to the data obtained for the obligate intracellular group, while the blue and violet curves correspond to the fitted logistic model to the data obtained for the extracellular and CA groups, respectively. The two *Mycoplasma* were removed from the CA groups for this analysis (see Methods).

As there is a clear trend in the INTRA group indicating that common reactions decrease to none rapidly, it is important to address the question whether the other groups follow the same rule. This analysis is based on a simulation of the number of common reactions for different subset sizes of the organisms (Figure 7). The exponential model fitted best the data of the INTRA group, however it did not fit well the data of the CA (without the *Mycoplasma* species, see Methods) and EXTRA groups (results not shown). We therefore tested a logistic model, which has a smoother decay than the exponential model, and found that it fitted much better this data. As expected, the asymptote for the INTRA group (*α*_*INTRA*_) was not significantly different from zero (Table 4). In contrast, the asymptote was estimated at 54 for the CA and 66 for the EXTRA. Thus, based on the analysed dataset, neither the CA nor the EXTRA group is expected to have an empty common set of reactions.

**Table 4:** Parameters estimated for the fitting models

**Lifestyle group (*l*)**	**Model**	***α*_*l*_**	***r*_*l*_**	***N*_*l*_**
INTRA	Exponential	1.70±5.17	0.56±0.19	269.48±122.30
CA	Logistic	53.84±4.37	0.06±0.01	1643.00±193.08
EXTRA	Logistic	65.85±3.06	0.06±0.00	1748.00±137.10

Estimates were obtained by fitting the exponential and logistic models to the corresponding data sets. *α*_*l*_is the asymptote, *r*_*l*_is the decay rate and *N*_*l*_is theoretically defined as the mean of the reaction sets for an empty subset size of the *l*^*th*^lifestyle group. Point estimates are given with ±2× standard error.

#### Core enzymatic function based on an EC number analysis for lifestyle groups

The number of shared partial EC numbers depends on the lifestyle groups: 7 were found for the INTRA, 28 for the CA and 52 for the EXTRA (Figure 6A and Additional file 7). These values represent 6%, 20% and 34% of the respective union in each group. The common set for the INTRA and CA groups is exactly the same as for the whole set of bacteria. The set for the INTRA group adds 3 more partial EC numbers when compared to the common partial EC numbers of the whole dataset: one oxidoreductase (1.5.1) and 2 transferases (2.1.1 and 2.6.1) (Figure 6B). The MIV bacteria are the ones which mainly account for the small size of the intersections in the INTRA group, and they share 8 partial EC numbers which means adding the ligase 6.3.5 to the common set. Hence, the common set of EC numbers for the MIV group comprises all classes of EC numbers, except for isomerase. Both the CA and the EXTRA shared sets have all the 6 classes of enzymes; the number of different EC numbers of each class ranges from two subclasses of lyases to 17 subclasses of transferases.

#### Connectivity of the partial EC number set for obligate intracellular and extracellular bacteria

We searched for connected reactions corresponding to the common partial EC numbers for the INTRA and EXTRA groups (7 and 52, respectively). We found no occurrence of connected reactions in either group (see Additional file 1 for a detailed description). Hence, the common set of partial EC numbers in the intracellular and EXTRA groups does not correspond to a connected portion of the metabolic network of these bacteria.

#### Differential random loss of enzymes

The differential random loss of enzymes or of biochemical capabilities (meaning partial EC numbers at level 3) was compared with the small intersection size of the partial EC number sets. Comparing the simulated values with the ones of the real MIV *Gammaproteobacteria* (Table 5 and Additional file 10), we found that the MIV have lost a greater diversity of biochemical capabilities than expected by simulation (Monte Carlo test, *p*<0.001).

**Table 5:** **Partial EC number sets for the MIV *Gammaproteobacteria *and for the simulated MIV**

	**Mean**	**Union**	**Intersection**
MIV *Gammaproteobacteria*	59	81	20
Differential random loss of reactions	69 ± 2.9	122 ± 6.2	21 ± 4.9
Estimated *p*−*value*	≤ 0.001	≤ 0.001	≤ 0.692
Differential random loss of biochemical capabilities	59 ± 0	114 ± 6.1	13 ± 4.4
Estimated *p*−*value*	1	≤ 0.001	≤ 0.006

Size of the mean, union and intersections of the sets of partial EC number for the MIV *Gammaproteobacteria* and for the simulated MIV.

#### Metabolites potentially acquired from the environment

The absence of a metabolic core in the INTRA might be linked to the differences in their environment. The number of metabolites that each bacterium potentially acquires from its environment (*i.e.*, potential inputs) ranged from 29 in “*Candidatus* Sulcia muelleri” (SULMW) to 341 in *M. smegmatis* (MYCS2) with a mean of 133 (Additional file 11). There are no potential inputs common to the 58 bacteria and the union of inputs is 1191 (Additional file 12). The intersection is null in the INTRA and the CA groups, while it is 2 in the EXTRA group. These two inputs are isolated from the rest of the network, and are linked together by one reaction which is catalysed by an enzyme that accelerates the folding of proteins (by catalysing the *cis-trans* isomerisation) [42]. Overall, we found no common inputs to the whole metabolic network of EXTRA bacteria. The mean values of the inputs in each group are 39, 133 and 190, respectively. Taking into account classes of compounds, the intracellular bacteria have in common ions, cofactors and nucleosides as potential inputs, while the EXTRA add vitamins and carbohydrates.

When we allowed distance one from the topological precursors (see Methods for details), the number of common inputs increased inside the lifestyle groups that have less organisms, such as PIV. In the broader lifestyle groups, the number of shared inputs remained equal. The number of bacteria that has glucose as input increased from 3 to 40. Furthermore, the size of the intersection augmented between the groups, such as MEH and PEH.

Overall, we find that neither EXTRA nor INTRA symbionts exhibit a common core of input metabolites. The absence of such a core is an intuitive explanation for the absence of a metabolic core of degradation pathways: different metabolic environments imply different metabolic pathways. However, this observation alone does not explain the total lack of a metabolic core for the INTRA symbionts. In this case, the specificity of the symbiosis with the host has to be considered.

## Discussion

In this paper, we investigated to what extent there is any reaction common to a set of bacteria, including obligate intracellular symbionts, as well as the influence and the trend of each lifestyle group concerning shared reactions or biochemical capabilities. In order to do this, we considered 58 bacteria carefully selected to represent a wide range of lifestyles.

### Existence of a metabolic core

Previous studies have found small sets of common metabolic genes even when including bacteria with reduced genomes [1,7]. Based on that and on the fact that we analysed reactions instead of genes (partially addressing the issue of NOGD), we therefore expected to find a small core of functional capabilities. Our analyses of the small molecule metabolism of 58 bacteria revealed however that they share no reaction, 16 compounds and 4 partial EC numbers.

Even though there was no reaction common to all bacteria, we actually found one reaction (3.5.1.88-RXN, MetaCyc [42]) present in all the dataset except in *M. hyopneumoniae* (MYCHJ). It is catalysed by the hydrolase peptide deformylase (Def), which releases the formyl group from the N-terminal methionine residue of most nascent polypeptides [65], an obligatory step during protein maturation in eubacteria [66]. The absence of Def in this bacterium apparently leaves it unable to formylate Met-tRNAi [67], and it has been described as absent or nonessential in *Phytoplasma* sp. and *Mycoplasma arthritidis*[67,68]. For long, peptide deformylase was believed to be exclusively present in bacteria, however Giglione *et al.*[69] identified eukaryotic deformylases which were localized in the organelles only. In our dataset, even the symbiont with most reduced genome (“*Ca.* Hodgkinia cicadicola” (HODCD)) is potentially capable to code for this enzyme. Nevertheless, recently an even smaller cellular genome (approx. 139 base pairs and 121 protein-coding genes) of “*Candidatus* Tremblaya princeps” has been described [70] which is missing homologs for Def. The presence of this enzyme in almost the whole dataset is justified by the fact that it is mostly related to information processing which is expected to be among the minimal functions required for sustaining life [1,3,7,8,19].

Such small sets found raised the question whether they could be explained only by the (6 or 8) bacteria with the smallest genomes. These bacteria had a weak impact on the number of shared reactions, while they had a strong effect on the common partial EC number set. Removing them, the shared set increased to 12 reactions mainly involved in the synthesis of a cell wall precursor, which is not considered as an essential pathway [14] and is known to be absent or reduced in host-dependent bacteria [71,72]. Conversely, the common partial EC number set increased to 30 without those bacteria which is a quite broad set of biochemical capabilities. All six classes of enzymes are included in this set, and are similar to the ones described for a minimal metabolism [21]. Only two partial EC numbers at level 3 (2.4.2 and 1.17.4) from this minimal metabolism are not included in our partial EC number set, however the latter partial EC number should not be in our analyses because it involves macromolecules and we work strictly with the small molecule metabolism. Furthermore, 8 of the 30 shared partial EC numbers are not included in this minimal metabolism, and four of them are transferases which are enriched in our common partial EC number set (43%).

The reduced set of common partial EC numbers raised the question whether it could be simply explained by a differential random loss of enzymes. This was not the case. We further identified the MIV *Gammaproteobacteria* as having lost a greater diversity of biochemical capabilities. This indicates that there is a set of partial EC numbers (capabilities) which are kept in subsets of organisms (not in every bacteria, *i.e.* it is not included in the shared set) and accounts for a reduced union.

Hence, we did not find a core of metabolic reactions shared by the symbiotic bacteria which agrees with the idea that searching for ubiquity as more genomes are included may ultimately reduce to nothing [20]. Conversely, using a more relaxed approach we found a core of biochemical capabilities which is similar to a minimal metabolism previously described [21].

### Impact of the lifestyle groups on the existence of a metabolic core

Among the different types of classification that we considered – (i) obligate intracellular, extracellular, cell associated, (ii) mutualistic, commensalist, parasitic, (iii) vertically or horizontally transmitted – the first is by far the one that explains best the differences in terms of metabolism. The CA group also accounted for the small common sets exclusively because of the *Mycoplasma* species. Even if this group presents other host-dependent bacteria, their genome sizes at least double when compared to the *Mycoplasma* species, and a core of reactions similar in size to the EXTRA is found. The other lifestyle groups (EXTRA and FL), which include just free-living bacteria, did not contribute to the size of the common set.

Furthermore, the impact of the INTRA and of the *Mycoplasma* species in the small sets can be directly related to their extremely reduced genomes [73-75]. They also have much fewer metabolic genes, even though this category is much less affected by the reduction in the INTRA group specially in the MIV. These bacteria (except for *W. pipientis* wBm (WOLTR)) are the most integrated [76] and are those for which the association with the host is essentially nutritional [25,57-64]. Indeed, the ratio of metabolic genes is significantly higher for MIV, indicating that the loss of genes primarily concerns the non metabolic ones [71,77,78]. The loss of metabolic genes is affected by the requirements for host survival, and to some extent by the presence of other symbionts in the same environment [71].

### Content and connectivity of the core metabolism of CA and EXTRA

In the analyses of each lifestyle group, we did not find a core of reactions for the INTRA, however we found it for the EXTRA and CA (the latter group without the two *Mycoplasma* species - the CA mentioned henceforward is without these bacteria). The shared reactions are involved in metabolic pathways that are also included in the minimal metabolism described by [8,21], such as glycolysis and nucleotide biosynthesis. The cores found also include amino acid biosynthesis pathways which are not present in the minimal metabolism because they assumed a nutrient-rich medium with amino acids unlimitedly available for the minimal cell [8,21].

The common sets of reactions of the CA and EXTRA groups are enriched in biosynthesis (approx. 88%) according to the metabolic processes defined in the BioCyc databases. In the core metabolism of *E. coli*, biosynthetic reactions are also overrepresented (57%) [32], thus our study enables to confirm and extend this result to multiple species. Overall, the core-metabolism of the CA and EXTRA bacteria is therefore much smaller than the one of the strains of *E. coli*, but at the same time, it is even more enriched in biosynthetic reactions. The reason for such an enrichment could be that, while the needs of the CA and the EXTRA symbionts are very similar in terms of building blocks for protein and DNA synthesis, the nutrients they uptake in their respective environment may be extremely variable. When variable environments are considered, degradation pathways, which are closer to the inputs of the network, are the first to be modified. This explanation is also corroborated by our observations on the lack of common inputs to all bacteria.

Considering now the proportion of biosynthesis and degradation reactions in the variable metabolism, we find that it is quite similar in *E. coli* (36% biosynthesis and 35% degradation) and the CA and EXTRA bacteria (approx. 39% biosynthesis and approx. 35% degradation), but the numbers are quite different for obligate intracellular bacteria (62% biosynthesis and 24% degradation). A possible explanation for this is that degradation pathways have largely disappeared in obligate intracellular bacteria, as the host provides an interface between the environment and the bacterium, while synthetic routes have not all disappeared but have been selected for, depending on the nature of the symbiosis [71,75,77,78].

Here, we worked with whole metabolic networks enabling to check whether the metabolic core would represent chains of biochemical reactions regardless of specific metabolic pathways. The core of reactions found was not entirely connected, most likely because of the existence of alternative pathways as highlighted by Gil *et al.*[8]. This means that searching for ubiquity even inside lifestyle groups does not result in one functional metabolic network.

### Persistent metabolic core of CA and EXTRA

We found a core of metabolic reactions for the CA and EXTRA, however we did not find one for the INTRA. This raised the question whether, as we add organisms, the decay of shared reactions and its limit was the same in these groups. First, we fitted the exponential model with asymptote to the data of all groups. This model described well the decay of shared reactions in the INTRA group. However, it was not appropriate to fit the EXTRA and CA data, since their behaviour of decay was not the same as that for the INTRA. Conversely, the logistic model was well adapted for these two groups. We also tested for common parameters for the two groups, but model fitting was better with each group having its separate parameter values. The decay rates (*r*_*CA*_ and *r*_*EXTRA*_) were similar, while the two other parameters were different. In principle we cannot give a direct biological interpretation to *N*_*l*_(it corresponds to the mean of the reaction sets for an empty subset size of organisms), we found its estimates are close to the size of the union of reactions of the corresponding lifestyle group, *e.g.*, *N*_*EXTRA*_ was estimated at 1643, while the size of the union of EXTRA was 1725 reactions. As expected, the asymptote estimated for the INTRA was not significantly different from zero, which agrees with the absence of a core of metabolic reactions found for this group. Conversely, the asymptotes estimated for the CA and the EXTRA groups were significantly different from zero; thus, based on the analysed dataset, neither group is expected to have an empty common set of reactions when more genomes of these groups are added. One should be aware that adding one organism that has a very particular niche could certainly change this trend. This result is nevertheless interesting given the fact that there are organisms from distinct taxonomic classes inside these groups, that moreover present different types of association with their hosts. To have an idea of the subset of reactions that would be “asymptotically” kept in organisms with lifestyles similar to those two groups, we analysed the reactions shared by the EXTRA and CA groups in our dataset. These 62 reactions are involved in the synthesis of purine and pyrimidine, of peptidoglycan and glycolysis. These findings are similar in number of enzymatic steps and in the content of pathways to the minimal metabolism described by Gabaldón *et al.*[21].

## Conclusions

In this paper, we explored to which extent each lifestyle group contributes to the reduction of a core metabolism as well as the composition of this core in the different groups, with a special focus on bacterial species only, in particular those that entertain a symbiotic relationship with a host. Moreover, we considered reactions instead of genes. Although we might then have expected to find a core, none common to all bacteria was observed. Symbionts with the most reduced genomes in our dataset had a weak impact on the number of shared reactions, but had a strong effect on the common partial EC number set which increased to 30 without those bacteria, covering a quite broad set of biochemical capabilities similar to those described for a minimal metabolism, with however an enrichment in transferases.

Obligate intracellular symbionts appeared as the main reason for such absence of a core of metabolic reactions due to their high specialisation. However, hostdependence alone is not an explanation for this absence. Indeed, although the cell associated group contained host-dependent bacteria, their core of reactions was observed to be similar in size to the one of extracellular bacteria once the two *Mycoplasma* species were eliminated from the group. Extremely reduced genomes such as those of the two *Mycoplasma* and of the intracellular group remain thus the main factor behind the absence of a core, even though the loss of genes primarily concerns the non metabolic ones.

A core of reactions was found for the cell-associated and the extracellular bacteria. This core roughly corresponds to the minimal metabolism previously described in the literature. It is not entirely connected and therefore does not result in one functional metabolic network. Although smaller than the core previously identified for strains of *E. coli*, we observed that it is even more enriched in biosynthetic reactions, which might be due to the extreme variability of the nutrients that cell-associated and extracellular bacteria uptake in their respective environment. On the other hand, the proportion of biosynthesis and degradation reactions in the variable metabolism appears quite similar to the one found in *E. coli*. The same is not the case for obligate intracellular bacteria where degradation pathways have largely disappeared but synthetic routes appear instead to have been selected for depending on the nature of the symbiosis.

Finally, by using simulation, we tested whether the decay of shared reactions and its limit would be the same for cell-associated and extracellular bacteria as for the intracellular ones. Although one should be aware that adding one organism that has a very particular niche could certainly change the result observed, it appears that a subset of around 60 reactions would be “asymptotically” kept in cell-associated and extracellular bacteria. These are involved in the synthesis of purine and pyrimidine, of peptidoglycan and glycolysis, and are similar in number of enzymatic steps and content of pathways to the minimal metabolism described in the literature.

## Competing interests

The authors declare that they have no competing interests.

## Author’s contributions

CCK selected the dataset and LC prepared the data. CCK conducted the computational analyses described in the paper. JK and CCK performed the simulation of the decay of the reaction sets. All authors contributed equally to coordinating the analyses and writing the manuscript. All authors read and approved the final manuscript.

## Supplementary Material

Additional file 1 **Additional results.** Additional results exemplifying the issue of NOGD, controlling for the structuring ofMIV and connectivity of the partial EC number set for obligate intracellular and extracellular bacteria. Click here for file

Additional file 2 **The full list of the bacteria selected and their detailed classification.** Additional file 2: Table S1: the full list of the bacteria selected and their detailed classification Click here for file

Additional file 3 **Number of Genes vs lifestyles.** Additional file 3: Figure S1: Total number of genes and number of metabolic genes (small bars) according to lifestyles (A) and taxonomic classes (B). Click here for file

Additional file 4 **Compounds common to all dataset.** Additional file 4: Table S2: compounds common to all dataset and their classification. Click here for file

Additional file 5 **Compounds and reactions common to groups of lifestyle.** Additional file 5: Table S3: Size of the mean, union and intersections of the compound and the reaction sets among the different lifestyle groups of bacteria. Click here for file

Additional file 6 **The full list of the reactions analysed and the list of organisms that lack them.** Additional file 6: Table S4: The full list of reactions represented by its MetaCyc ID, corresponding enzyme name and EC number. For each reaction, the number of organisms that possess it are presented as well as the list of the bacteria which lack it. The codes for the organisms are the ones from HAMAP [ [40]] and they can also be found in Figure 1 and in the Additional file 1. The reactions are sorted by the number of organisms that possess them. Click here for file

Additional file 7 **Partial EC numbers common to groups of lifestyle.** Additional file 7: Table S5: size of the mean, union and intersections of the partial EC number sets among the different lifestyle groups. Click here for file

Additional file 8 **Metabolic core of the extracellular bacteria.** Additional file 8: Figure S2: qualitative representation of the metabolic core of the extracellular symbionts. Click here for file

Additional file 9 **Distribution of reactions across metabolic processes.** Additional file 9: Table S6: distribution of reactions across metabolic processes, as defined in BioCyc databases. Some reactions are classified in more than one process and this explains why it may happen that the two proportions add up to more than 100%. For clarity, we introduced a category for such reactions (Biosynthesis/Degradation). For the group CA, the values in-between parentheses correspond to the CA group without the two *Mycoplasma* species. Click here for file

Additional file 10 **Simulation of partial EC number sets of the MIV bacteria.** Simulation of partial EC number sets of theMIV bacteria. Additional file 10: Figure S3: simulation of the size of the mean (A), union (B) and intersection (C) of the partial EC number sets of the MIV bacteria. Click here for file

Additional file 11 **Number of potential inputs.** Additional file 11: Figure S4: the number of potential inputs of the metabolic networks according to Borenstein method [ [52]]. Click here for file

Additional file 12 **Potential inputs common to groups of lifestyle.** Additional file 12: Table S7: size of the mean, union and intersections of the potential input sets among the different lifestyle groups. Click here for file
