# Supplementary material for: Exploration of the core metabolism of symbiotic bacteria
Source: BMC Genomics. 2012 Aug 31;13:438. doi: 10.1186/1471-2164-13-438 (PMC3543179; doi:10.1186/1471-2164-13-438)
Supplement: Additional file 1 — Additional results. Additional results exemplifying the issue of NOGD, controlling for the structuring ofMIV and connectivity of the partial EC number set for obligate intracellular and extracellular bacteria. [file 1471-2164-13-438-S1.pdf]

Additional results

Exemplifying the issue of NOGD

We partially deal with NOGD because different orthologous families encoding one enzymatic capability often turn out to be a same reaction. One such example is an intermediate enzyme in the glycolytic pathway phosphoglycerate mutase (pgm), which has no sequence similarity between *Mycoplasma genitalium* and *Haemophilus influenzae* [1], and is represented by only one enzymatic reaction in our dataset. In fact, we have both analogous enzymes in our dataset: the cofactor-independent pgm (*e.g.*, *M. genitalium*, *Agrobacterium*, *Pseudomonas*) and the cofactor-dependent pgm (*e.g.*, *Streptococcus*, *Bartonella*, *Buchnera*) based on the analysis of Foster *et al.* [2]. The reasoning for only one reaction representing both analogous enzymes is a many-to-many correspondence of enzyme-reaction, *i.e.*, one protein may catalyse more than one reaction, while the same reaction may be catalysed by more than one protein, and these multiple proteins catalysing a same reaction may or may not show sequence homology [3]. Moreover, one should be aware that some cases of NOGD do not result in only one reaction, such as thymidylate synthase which is folate-dependent (ec:2.1.1.45) in *M. genitalium* and in most bacteria, while it is flavin-dependent (ec:2.1.1.148) in *Actinobacteria*, *Rickettsia* and *Chlamydia* [4]. These enzymatic capabilities are classified with different EC numbers at level 4, however in our analysis of partial EC numbers at level 3, they are classified in the same way (2.1.1).

Controlling for the structuring of MIV

As mentioned, we found that the absence of a metabolic core common to all symbionts is mainly caused by the absence of such a core within the MIV group. The multiple correspondence analysis we performed to further explore the structure of this group resulted in two subgroups of MIV symbionts which present opposite patterns of reaction presence/absence (Figure 1 in this document). Subgroup A presented mainly reactions involved in the biosynthesis of amino acids, whereas subgroup B showed reactions involved in heme synthesis. Furthermore, these reactions can be directly related to the role of the symbiont in the mutualistic relationship. Indeed, *Wigglesworthia glossinidia* (WIGBR) and *Wolbachia pipientis* wBm (WOLTR) are known to supply heme to their hosts [5–7], while "*Candidatus* Sulcia muelleri" (SULMW) and *Buchnera aphidicola* APS (BUCAI) are known to provide amino acids [8–10]. In the case of *Candidatus* Blochmannia floridanus (BLOFL), *Candidatus* Blochmannia pennsylvanicus (BLOPB) and *Blattabacterium* sp. Bge (BLASB), the main symbiotic function is the metabolism of nitrogen, but the conservation of most of the pathways for the synthesis of essential amino acids indicates that they may also have an important role in the symbiosis

[6, 11–13].

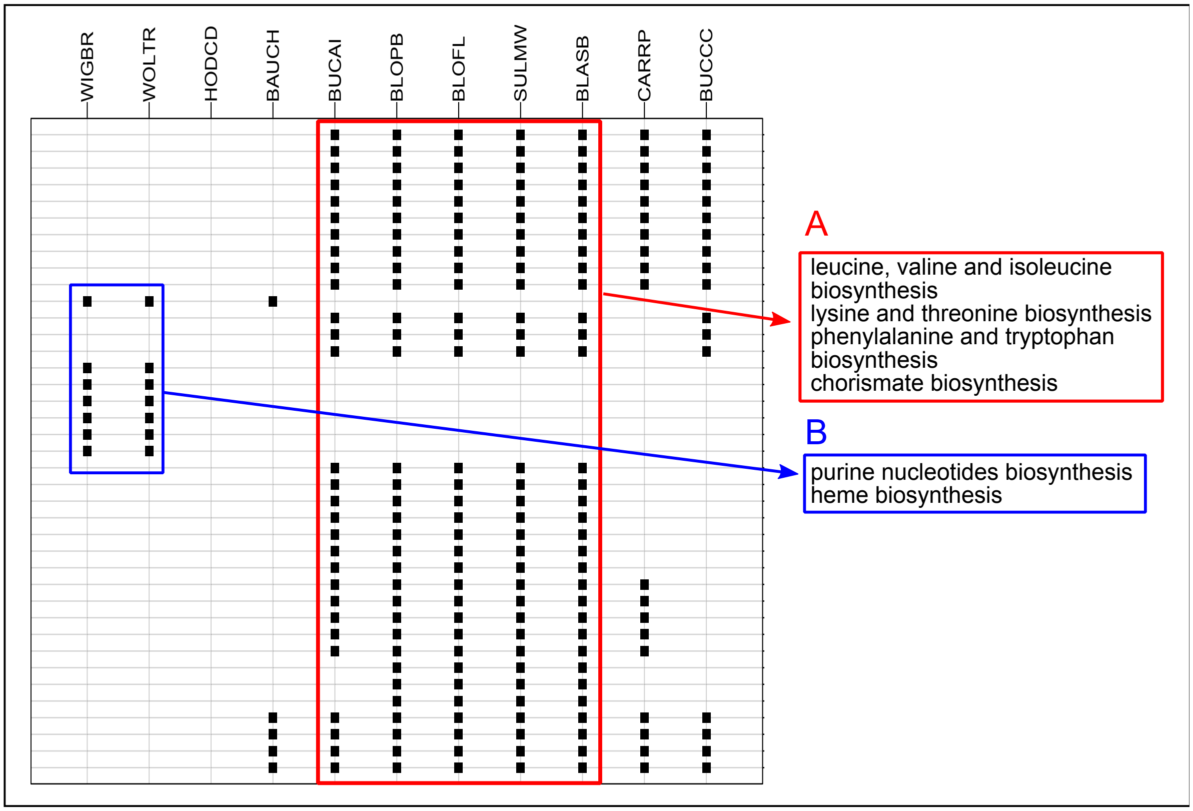

Figure 1: Two subgroups of MIV symbionts which present opposite patterns of reaction presence/absence. Table of the presence/absence of reactions with correlation ratio greater than 50% on the second axis of the MCA. The square indicates the presence of the reaction in the organism of the respective column. The red rectangle indicates subgroup A: *Buchnera aphidicola* APS (BUCAI), "*Candidatus* Blochmannia pennsylvanicus" (BLOPB), "*Candidatus* Blochmannia floridanus" (BLOFL), "*Candidatus* Sulcia muelleri" (SULMW) and *Blattabacterium* sp. Bge (BLASB) which presented mainly reactions involved in the biosynthesis of amino acids. The blue rectangle indicates subgroup B: *Wigglesworthia glossinidia* (WIGBR) and *Wolbachia pipientis* wBm (WOLTR) which showed reactions involved in heme synthesis.

Other than the impact of the small networks, this structuring of the MIV symbionts in two subgroups could also have an impact on the size of the intersection of the reaction sets. To test that, we calculated the intersection of the reaction sets between each subgroup (A or B) and the other bacteria which are not in the MIV group (47 organisms). This resulted in null for group A and 5 reactions for group B. Removing the two species of *Mycoplasma*, the intersection sizes are 2 reactions for the group A plus the non MIV bacteria and 27 for the group B. As the size of the intersection remains small in either subgroup of MIV symbionts with the non MIV organisms, the structuring of the MIV symbionts does not explain the reduced number

of reactions shared by the bacteria analysed.

Connectivity of the partial EC number set for obligate intracellular and extracellular bacteria

As mentioned above, the size of the shared set of partial EC numbers for the INTRA is 7 while for the EXTRA it is 52. The 7 partial EC numbers common to the intracellular bacteria correspond to 154 reactions in the union of all reactions of this group, whereas the 52 of the EXTRA bacteria correspond to 1253 reactions. The same procedure of searching for connected reactions corresponding to the common partial EC numbers in the INTRA and EXTRA groups was performed in the union reaction graph of each group. The induced reaction graph from the 154 reactions in the intracellular group is composed of 174 arcs and 19 connected components apart from 47 isolated reactions. There is no connected set of 7 reactions labelled with the 7 partial EC numbers common to the intracellular symbionts. In the case of the EXTRA group, the induced reaction graph from the 1253 reactions has 5288 arcs and 38 connected components apart from 181 isolated reactions. There is no occurrence of 52 reactions whose EC numbers are the 52 common to the EXTRA bacteria that is connected in this graph. Hence, the common set of partial EC numbers in the intracellular and EXTRA groups does not correspond to a connected portion of the metabolic network of these bacteria.

Additional methods

Controlling for the structuring of MIV

In order to further explore the structure of MIV (Mutualistic Intracellular Vertically transmitted, see Figure 1 for abbreviations of group names) as they have the smallest genome sizes of the dataset and they have specific symbiotic functions, we performed a multiple correspondence analysis (MCA) using the R [14] package ADE4 [15]. The input data was the contingency table of the presence and absence of reactions for each organism. We analysed the reactions with correlation ratio greater than 85% and 50% on the first and second axis (respectively) of the MCA.

References

1. Koonin EV, Mushegian AR, Bork P: **Non-orthologous gene displacement.** *Trends in genetics : TIG* 1996, **12**(9):334–336, [http://view.ncbi.nlm.nih.gov/pubmed/8855656].
2. Foster JM, Davis PJ, Raverdy S, Sibley MH, Raleigh EA, Kumar S, Carlow CK: **Evolution of bacterial phosphoglycerate mutases: non-homologous isofunctional enzymes undergoing gene losses, gains and lateral transfers.** *PloS one* 2010, **5**(10), [http://dx.doi.org/10.1371/journal.pone.0013576].
3. Karp PD, Riley M: **Representations of metabolic knowledge.** *Proceedings / ... International Conference on Intelligent Systems for Molecular Biology ; ISMB. International Conference on Intelligent Systems for Molecular Biology* 1993, **1**:207–215, [http://view.ncbi.nlm.nih.gov/pubmed/7584337].
4. Koonin EV: **Comparative genomics, minimal gene-sets and the last universal common ancestor.** *Nature reviews. Microbiology* 2003, **1**(2):127–136.
5. Akman L, Yamashita A, Watanabe H, Oshima K, Shiba T, Hattori M, Aksoy S: **Genome sequence of the endocellular obligate symbiont of tsetse flies, Wigglesworthia glossinidia.** *Nature genetics* 2002, **32**(3):402–407.
6. Zientz E, Dandekar T, Gross R: **Metabolic interdependence of obligate intracellular bacteria and their insect hosts.** *Microbiology and molecular biology reviews : MMBR* 2004, **68**(4):745–770.
7. Foster J, Ganatra M, Kamal I, Ware J, Makarova K, Ivanova N, Bhattacharyya A, Kapatral V, Kumar S, Posfai J, Vincze T, Ingram J, Moran L, Lapidus A, Omelchenko M, Kyrpides N, Ghedin E, Wang S, Goltsman E, Joukov V, Ostrovskaya O, Tsukerman K, Mazur M, Comb D, Koonin E, Slatko B: **The Wolbachia genome of Brugia malayi: endosymbiont evolution within a human pathogenic nematode.** *PLoS biology* 2005, **3**(4).
8. McCutcheon JP, Moran NA: **Parallel genomic evolution and metabolic interdependence in an ancient symbiosis.** *Proceedings of the National Academy of Sciences of the United States of America* 2007, **104**(49):19392–19397.
9. Baumann P, Baumann L, Lai CY, Rouhbakhsh D, Moran NA, Clark MA: **Genetics, physiology, and evolutionary relationships of the genus Buchnera: intracellular symbionts of aphids.** *Annual review of microbiology* 1995, **49**:55–94.
10. Shigenobu S, Watanabe H, Hattori M, Sakaki Y, Ishikawa H: **Genome sequence of the endocellular bacterial symbiont of aphids Buchnera sp. APS.** *Nature* 2000, **407**(6800):81–86.
11. Gil R, Silva FJ, Zientz E, Delmotte F, González-Candelas F, Latorre A, Rausell C, Kamerbeek J, Gadau J, Hölldobler B, van Ham RC, Gross R, Moya A: **The genome sequence of Blochmannia floridanus: comparative analysis of reduced genomes.** *Proceedings of the National Academy of Sciences of the United States of America* 2003, **100**(16):9388–9393.
12. Degnan PH, Lazarus AB, Wernegreen JJ: **Genome sequence of Blochmannia pennsylvanicus indicates parallel evolutionary trends among bacterial mutualists of insects.** *Genome research* 2005, **15**(8):1023–1033.
13. López-Sánchez MJ, Neef A, Peretó J, Patiño Navarrete R, Pignatelli M, Latorre A, Moya A: **Evolutionary convergence and nitrogen metabolism in Blattabacterium strain Bge, primary endosymbiont of the cockroach Blattella germanica.** *PLoS genetics* 2009, **5**(11):e1000721+.
14. R Development Core Team: *R: A Language and Environment for Statistical Computing.* R Foundation for Statistical Computing, Vienna, Austria 2009.
15. Dray S, Dufour A: **The ade4 package: implementing the duality diagram for ecologists**. *Journal of Statistical Software* 2007, **22**(4):1–20.
