# Supplementary material for: Exploration of the core metabolism of symbiotic bacteria
Source: BMC Genomics. 2012 Aug 31;13:438. doi: 10.1186/1471-2164-13-438 (PMC3543179; doi:10.1186/1471-2164-13-438)
Supplement: Additional file 2 — The full list of the bacteria selected and their detailed classification. Additional file 2: Table S1: the full list of the bacteria selected and their detailed classification [file 1471-2164-13-438-S2.pdf]

Table S1: Dataset classification

ID	Name	Number of Genes	Taxonomic class	Type of Association	Obligatoriness of the association for the bacterium	for the host	Location	Transmission
HODCD	Candidatus Hodgkinia cicadicola Dsem	203	ALP	M	O	P	I	V
CARRP	Candidatus Carsonella ruddii PV	214	GAM	M	O	P	I	V
SULMW	Candidatus Sulcia muelleri GWSS	266	FLA	M	O	P	I	V
BUCCC	Buchnera aphidicola Cc	396	GAM	M	O	P	I	V
MYCGE	Mycoplasma genitalium G37	553	MOL	P	O	S	CA	H
BUCAI	Buchnera aphidicola APS	619	GAM	M	O	P	I	V
BLASB	Blattabacterium sp. Bge	638	FLA	M	O	P	I	V
BAUCH	Baumannia cicadellinicola	657	GAM	M	O	P	I	V
BLOFL	Candidatus Blochmannia floridanus	668	GAM	M	O	P	I	V
WIGBR	Wigglesworthia glossinidia	671	GAM	M	O	P	I	V
BLOPB	Candidatus Blochmannia pennsylvanicus	718	GAM	M	O	P	I	V
MYCHJ	Mycoplasma hyopneumoniae J	754	MOL	P	O	S	CA	H
RICTY	Rickettsia typhi Wilmington	976	ALP	P	O	S	I	V
CHLTR	Chlamydia trachomatis D/UW-3/CX	987	CHL	P	O	S	I	H
WOLTR	Wolbachia pipientis wBm	1188	ALP	M	O	P	I	V
WOLPM	Wolbachia pipientis wMel	1416	ALP	P	O	S	I	V
BARQU	Bartonella quintana Toulouse	1433	ALP	P	O	S	CA	H
LAWIP	Lawsonia intracellularis PHE/MN1-00	1530	DEL	P	O	S	I	H
ORITB	Orientia tsutsugamushi Boryong	1580	ALP	P	O	S	I	V
HELPY	Helicobacter pylori 26695	1718	EPS	P	O	S	CA	H
STRTD	Streptococcus thermophilus LMD-9	1836	BAC	C	FA	S	E	H
NEIG2	Neisseria gonorrhoeae NCCP11945	1947	BET	P	O	S	CA	H
WOLSU	Wolinella succinogenes DSM 1740	2138	EPS	C	FA	S	E	H
STRA5	Streptococcus agalactiae 2603V/R	2160	BAC	P	FA	S	E	H
THICR	Thiomicrospira crunogena XCL-2	2353	EPS	FL	FL	FL	FL	FL
HAMD5	Candidatus Hamiltonella defensa T5A	2387	GAM	M	O	S	CA	V
LACC3	Lactobacillus casei ATCC 334	2949	BAC	C	FA	S	E	H
LISMO	Listeria monocytogenes EGD-e	3084	BAC	P	FA	S	CA	H
XYLFA	Xylella fastidiosa 9a5c	3261	GAM	P	FA	S	E	H
BRUME	Brucella melitensis bv 1 16M	3492	ALP	P	O	S	CA	H
DESPS	Desulfotalea psychrophila Lsv54	3600	DEL	FL	FL	FL	FL	FL
PSEHT	Pseudoalteromonas haloplanktis TAC125	3612	GAM	FL	FL	FL	FL	FL
VIBCH	Vibrio cholerae O1 biovar El Tor str. N16961	4025	GAM	P	FA	S	E	H
BACA2	Bacillus amyloliquefaciens FZB42	4033	BAC	C	FA	S	E	H
YERPE	Yersinia pestis CO92	4344	GAM	P	O	S	CA	H
BACSU	Bacillus subtilis 168	4353	BAC	FL	FL	FL	FL	FL
ECOLI	Escherichia coli K-12	4390	GAM	C	FA	S	E	H
YERPY	Yersinia pseudotuberculosis YPIII	4618	GAM	P	FA	S	CA	H
SODGM	Sodalis glossinidius morsitans	4649	GAM	M	O	S	CA	V
RHOS4	Rhodobacter sphaeroides 2.4.1	4672	ALP	FL	FL	FL	FL	FL
MYCTU	Mycobacterium tuberculosis H37Rv	4679	ACT	P	O	S	CA	H
SALTI	Salmonella enterica serovar Typhi	4712	GAM	P	FA	S	CA	H
SHIFL	Shigella flexneri 2a str. 301	4728	GAM	P	FA	S	CA	H
ERWCT	Erwinia carotovora subsp. atroseptica SCRI1043	4765	GAM	P	FA	S	E	H

continue on the next page

Table S1 – continue on the next page

ID	Name	Number of Genes	Taxonomic class	Type of Association	Obligatoriness of the association for the bacterium	for the host	Location	Transmission
PHOLL	Photorhabdus luminescens TTO1	4801	GAM	P	O	S	CA	H
BURMA	Burkholderia mallei ATCC 23344	5129	BET	P	O	S	CA	H
PSEE4	Pseudomonas entomophila L48	5241	GAM	P	FA	S	E	H
ECO57	Escherichia coli O157:H7	5549	GAM	P	FA	S	E	H
BACHK	Bacillus thuringiensis serovar konkukian str. 97-27	5599	BAC	P	FA	S	E	H
AGRT5	Agrobacterium tumefaciens C58	5700	ALP	P	FA	S	E	H
BACAN	Bacillus anthracis Ames Ancestor	5886	BAC	P	O	S	CA	H
CUPTR	Cupriavidus taiwanensis LMG19424	5940	BET	M	FA	S	E	H
PSEAB	Pseudomonas aeruginosa UCBPP-PA14	6254	GAM	P	FA	S	E	H
FRAAA	Frankia alni ACN14a	6763	ACT	M	FA	S	E	H
RHIME	Sinorhizobium meliloti 1021	6933	ALP	M	FA	S	E	H
PSEFS	Pseudomonas fluorescens SBW25	6960	GAM	C	FA	S	E	H
MYCS2	Mycobacterium smegmatis MC2 155	7223	ACT	C	FA	S	E	H
RALEH	Ralstonia eutropha H16	7279	BET	FL	FL	FL	FL	FL

Association: C, commensal; M, mutualist; P, parasite; FL, free-living. **Obligatoriness of the association for the bacterium**: FA, facultative; O, obrigatory. **Obligatoriness of the association for the host**: P, primary symbiont; S, secondary symbiont. **Location**: I, obligatory intracellular; CA: cell associated; E, extracellular. **Transmission**: H, horizontal; V, vertical.
