# Supplementary material for: Exploration of the core metabolism of symbiotic bacteria
Source: BMC Genomics. 2012 Aug 31;13:438. doi: 10.1186/1471-2164-13-438 (PMC3543179; doi:10.1186/1471-2164-13-438)
Supplement: Additional file 3 — Number of Genes vs lifestyles. Additional file 3: Figure S1: Total number of genes and number of metabolic genes (small bars) according to lifestyles (A) and taxonomic classes (B). [file 1471-2164-13-438-S3.pdf]

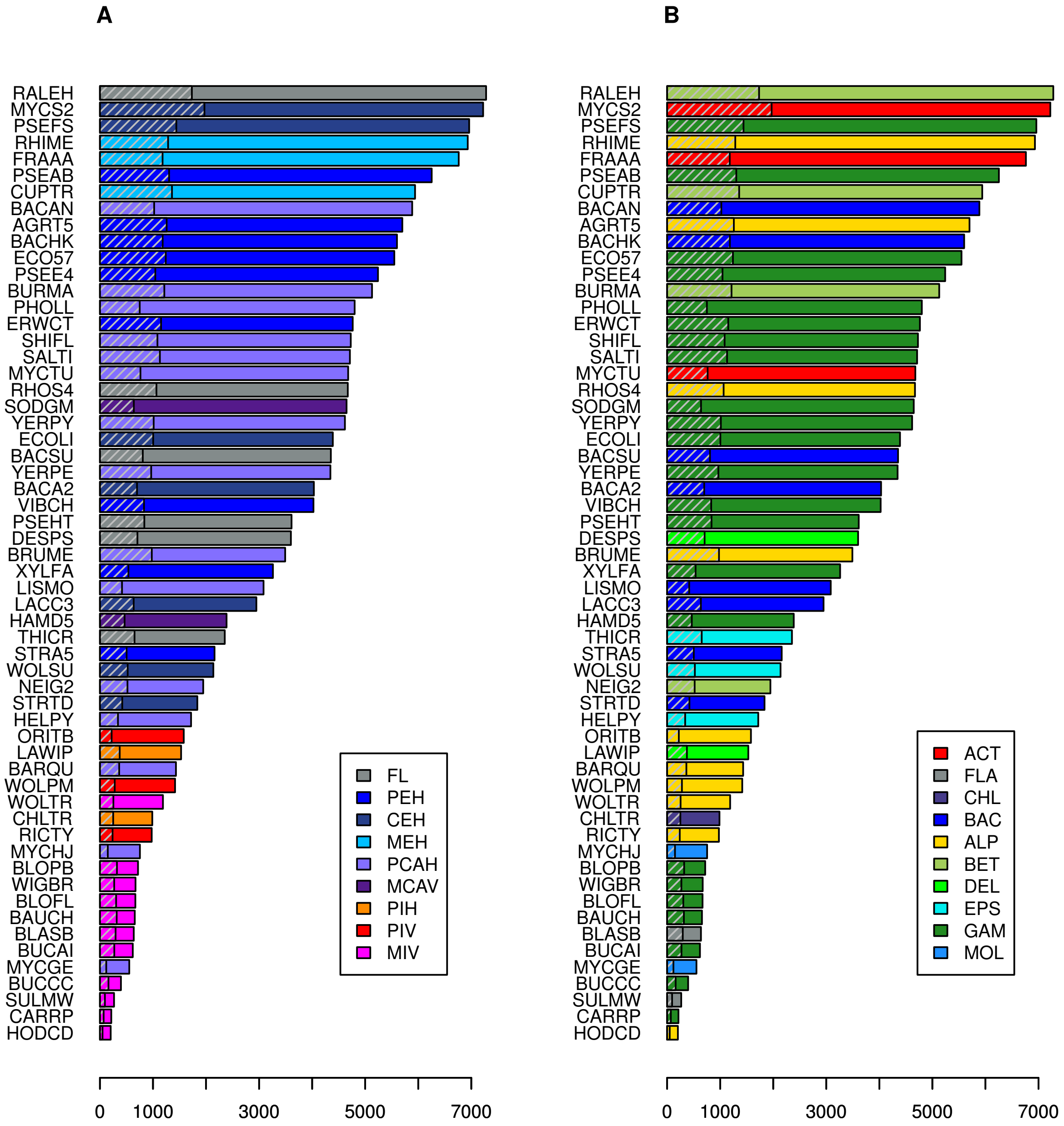

Figure S1: Total number of genes and number of metabolic genes (small bars) according to lifestyles (A) and taxonomic classes (B).
