# Supplementary material for: Exploration of the core metabolism of symbiotic bacteria
Source: BMC Genomics. 2012 Aug 31;13:438. doi: 10.1186/1471-2164-13-438 (PMC3543179; doi:10.1186/1471-2164-13-438)
Supplement: Additional file 4 — Compounds common to all dataset. Additional file 4: Table S2: compounds common to all dataset and their classification. [file 1471-2164-13-438-S4.pdf]

Table S2: **Compounds common to all dataset**

Compounds	Classification
pyruvate	Acids
L-alanine	Amino Acids
L-cysteine	Amino Acids
L-glutamate	Amino Acids
L-glutamine	Amino Acids
L-serine	Amino Acids
D-ribose-5-phosphate	Carbohydrates
coenzyme A	Cofactors
diphosphate	Ions
phosphate	Ions
H+ (PROTON)	Ions
AMP	Nucleosides
ADP	Nucleosides
ATP	Nucleosides
formate	Unclassified-Compounds
H2O (WATER)	Unclassified-Compounds
