# Supplementary material for: Exploration of the core metabolism of symbiotic bacteria
Source: BMC Genomics. 2012 Aug 31;13:438. doi: 10.1186/1471-2164-13-438 (PMC3543179; doi:10.1186/1471-2164-13-438)
Supplement: Additional file 5 — Compounds and reactions common to groups of lifestyle. Additional file 5: Table S3: Size of the mean, union and intersections of the compound and the reaction sets among the different lifestyle groups of bacteria. [file 1471-2164-13-438-S5.pdf]

Table S3: **Compounds and reactions common to groups of lifestyle**

		Compounds			Reactions		
	Nº Organisms	Mean (m)	Union (u)	Intersection (i)	Mean (m)	Union (u)	Intersection (i)
MIV	11	247	554	20	203	566	1
PIV	3	235	363	141	188	327	91
PIH	2	304	422	187	267	408	126
**Intra**	**16**	**252**	**647**	**19**	**209**	**705**	**1**
**% Intra**	**100%**	**m/u = 39%**	**i/m = 8%**	**i/u = 3%**	**m/u = 30%**	**i/m = 0.5%**	**i/u = 0.1%**
MCAV	2	502	656	348	493	682	304
PCAH	15	595	1491	67	598	1716	17
**CA**	**17**	**584**	**1496**	**67**	**585**	**1725**	**17**
**% CA**	**100%**	**m/u = 39%**	**i/m = 11%**	**i/u = 4%**	**m/u = 34%**	**i/m = 3%**	**i/u = 1%**
**Intra+CA**	**33**	**423**	**1513**	**16**	**403**	**1754**	**0**
**% Intra+CA**	**100%**	**m/u = 28%**	**i/m = 3.8%**	**i/u = 1.1%**	**m/u = 23%**	**i/m = 0%**	**i/u = 0%**
MEH	3	873	1227	562	877	1336	479
CEH	7	706	1557	221	706	1750	128
PEH	9	752	1520	254	773	1736	163
**Extra**	**19**	**754**	**1854**	**186**	**765**	**2143**	**94**
**% Extra**	**100%**	**m/u = 41%**	**i/m = 25%**	**i/u = 10%**	**m/u = 36%**	**i/m = 12%**	**i/u = 4%**
FL	6	706	1349	304	711	1548	202
**% FL**	**6**	**m/u = 52%**	**i/m = 43%**	**i/u = 23%**	**m/u = 46%**	**i/m = 28%**	**i/u = 13%**
**Extra+FL**	**25**	**743**	**1911**	**179**	**752**	**2232**	**84**
**% Extra+FL**	**100%**	**m/u = 39%**	**i/m = 24%**	**i/u = 9%**	**m/u = 34%**	**i/m = 11%**	**i/u = 4%**
**Total**	**58**	**561**	**1950**	**16**	**553**	**2285**	**0**
**% Total**	**100%**	**m/u = 29%**	**i/m = 2.9%**	**i/u = 0.8%**	**m/u = 24%**	**i/m = 0%**	**i/u = 0%**

Size of the mean, union and intersections of the compound and the reaction sets among the different lifestyle groups of bacteria.
