# Supplementary material for: Exploration of the core metabolism of symbiotic bacteria
Source: BMC Genomics. 2012 Aug 31;13:438. doi: 10.1186/1471-2164-13-438 (PMC3543179; doi:10.1186/1471-2164-13-438)
Supplement: Additional file 7 — Partial EC numbers common to groups of lifestyle. Additional file 7: Table S5: size of the mean, union and intersections of the partial EC number sets among the different lifestyle groups. [file 1471-2164-13-438-S7.pdf]

Table S5: **Partial EC numbers common to groups of lifestyle**

	Partial EC numbers			
	Mean	Union	Intersection	Intersection
MIV	55	99	8	1.5.1 2.1.1 2.3.1 2.5.1 2.6.1 3.5.1 4.2.1 6.3.5
PIV	59	83	39	
PIH	70	84	55	
Intra	58	108	7	1.5.1 2.1.1 2.3.1 2.5.1 2.6.1 3.5.1 4.2.1
% Intra	m/u=54%	i/m=12%	i/u=6%	
MCAV	86	94	77	
PCAH	91	143	28	
CA	90	143	28	
% CA	m/u=63%	i/m=31%	i/u=20%	
Intra+CA	75	144	4	2.3.1 2.5.1 3.5.1 4.2.1
% Intra+CA	m/u=52%	i/m=5%	i/u=3%	
MEH	111	131	89	
CEH	99	141	58	
PEH	106	140	60	
Extra	104	151	52	
% Extra	m/u=69%	i/m=50%	i/u=34%	
FL	105	142	70	
% FL	m/u=74%	i/m=67%	i/u=49%	
Extra+FL	104	154	50	
% Extra+FL	m/u=68%	i/m=48%	i/u=32%	
Total	87	156	4	2.3.1 2.5.1 3.5.1 4.2.1
% Total	m/u=56%	i/m=5%	i/u=3%	

Size of the mean, union and intersections of the partial EC number sets among the different lifestyle groups.
