# Supplementary figures and images for: Exploration of the core metabolism of symbiotic bacteria
Source: BMC Genomics. 2012 Aug 31;13:438. doi: 10.1186/1471-2164-13-438 (PMC3543179; doi:10.1186/1471-2164-13-438)

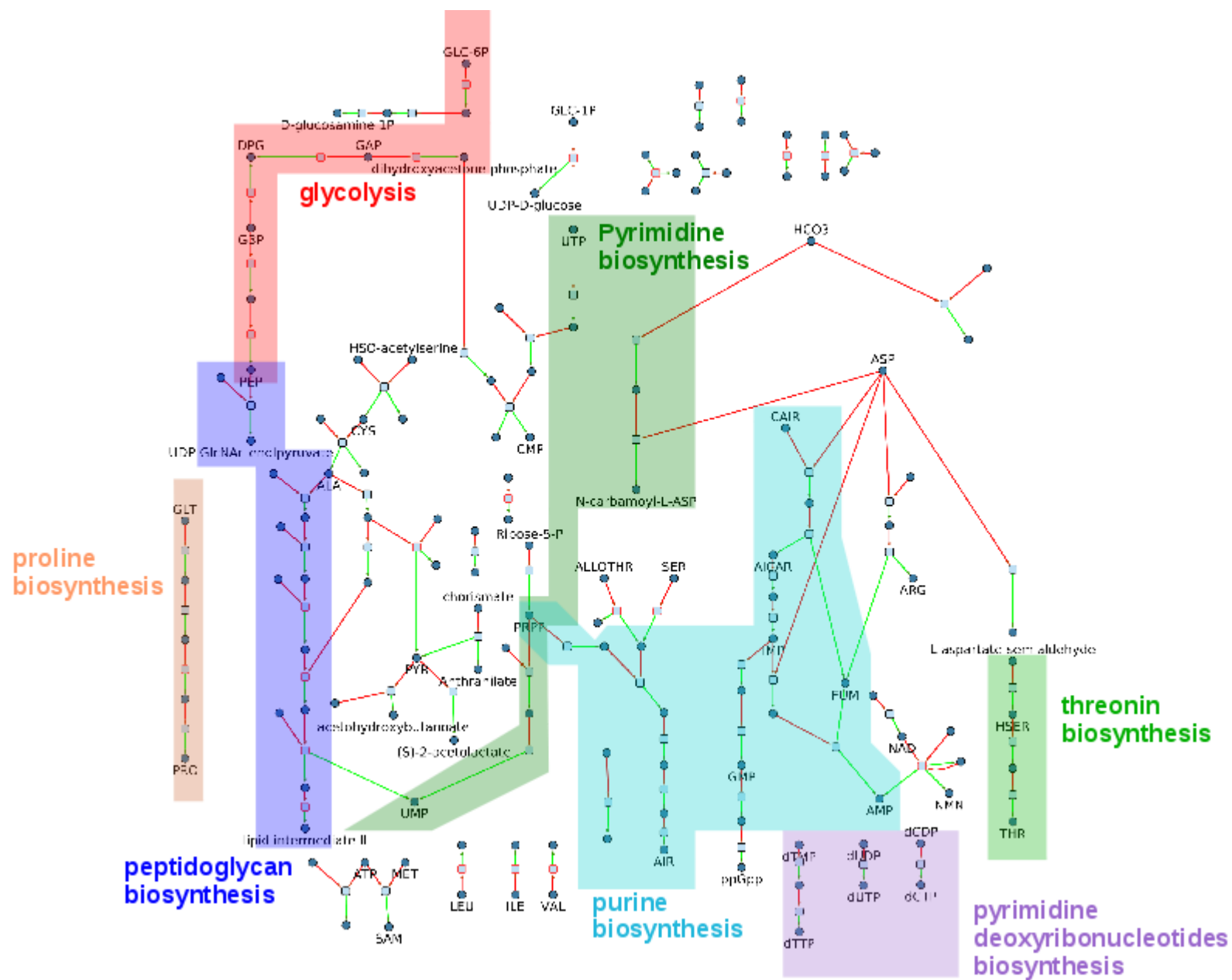

Figure S2: Qualitative representation of the metabolic core of the extracellular symbionts

Supplement: Additional file 8 — Metabolic core of the extracellular bacteria. Additional file 8: Figure S2: qualitative representation of the metabolic core of the extracellular symbionts. [file 1471-2164-13-438-S8.pdf]
