# Supplementary material for: Exploration of the core metabolism of symbiotic bacteria
Source: BMC Genomics. 2012 Aug 31;13:438. doi: 10.1186/1471-2164-13-438 (PMC3543179; doi:10.1186/1471-2164-13-438)
Supplement: Additional file 9 — Distribution of reactions across metabolic processes. Additional file 9: Table S6: distribution of reactions across metabolic processes, as defined in BioCyc databases. Some reactions are classified in more than one process and this explains why it may happen that the two proportions add up to more than 100%. For clarity, we introduced a category for such reactions (Biosynthesis/Degradation). For the group CA, the values in-between parentheses correspond to the CA group without the two Mycoplasma species. [file 1471-2164-13-438-S9.pdf]

Table S6: **Distribution of reactions across metabolic processes**

	Extracellular			CA			Intracellular
	core	variable	pan	core	variable	pan	variable
Biosynthesis	83	782	865	14 (78)	704 (640)	718 (718)	437
Degradation	24	722	746	9 (23)	581(567)	590 (590)	168
Biosynthesis/Degradation	24	136	160	9 (21)	130 (118)	139 (139)	86
Detoxification	1	7	8	0 (1)	7 (6)	7 (7)	5
Energy Metabolism	11	106	117	8 (16)	93 (85)	101 (101)	55
Transport	0	2	2	0 (0)	2 (2)	2 (2)	1
Total	94	2049	2143	17 (88)	1708 (1637)	1725 (1725)	704

Distribution of reactions across metabolic processes, as defined in BioCyc databases. Some reactions are classified in more than one process and this explains why it may happen that the two proportions add up to more than 100%. For clarity, we introduced a category for such reactions (Biosynthesis/Degradation). For the group CA, the values in-between parentheses correspond to the CA group without the two *Mycoplasma* species.
