# Supplementary material for: Exploration of the core metabolism of symbiotic bacteria
Source: BMC Genomics. 2012 Aug 31;13:438. doi: 10.1186/1471-2164-13-438 (PMC3543179; doi:10.1186/1471-2164-13-438)
Supplement: Additional file 10 — Simulation of partial EC number sets of the MIV bacteria. Simulation of partial EC number sets of theMIV bacteria. Additional file 10: Figure S3: simulation of the size of the mean (A), union (B) and intersection (C) of the partial EC number sets of the MIV bacteria. [file 1471-2164-13-438-S10.pdf]

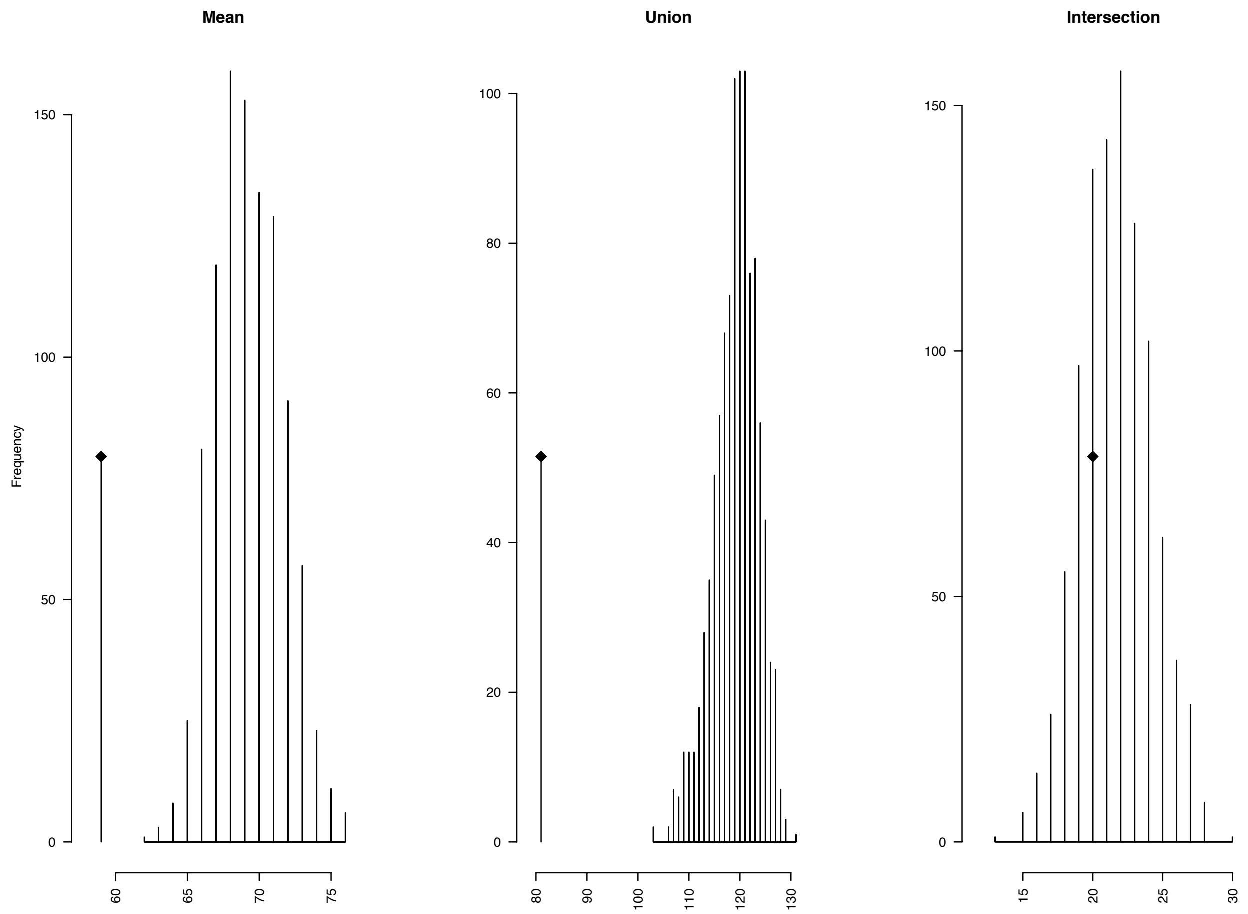

Figure S3: Simulation of the size of the mean (A), union (B) and intersection (C) of the partial EC number sets of the MIV bacteria.
