# Supplementary material for: Exploration of the core metabolism of symbiotic bacteria
Source: BMC Genomics. 2012 Aug 31;13:438. doi: 10.1186/1471-2164-13-438 (PMC3543179; doi:10.1186/1471-2164-13-438)
Supplement: Additional file 11 — Number of potential inputs. Additional file 11: Figure S4: the number of potential inputs of the metabolic networks according to Borenstein method [ [52]]. [file 1471-2164-13-438-S11.pdf]

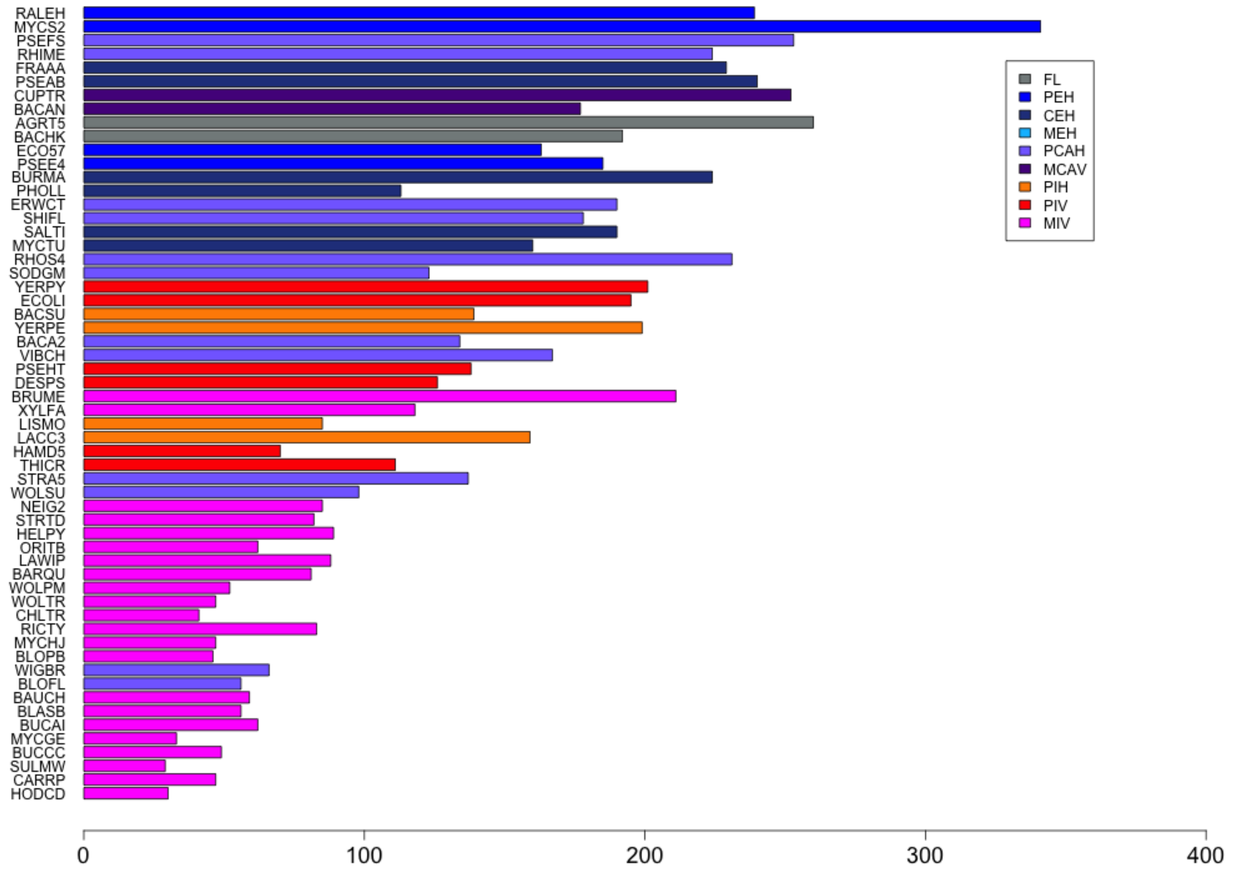

Figure S4: The number of potential inputs of the metabolic networks according to Borenstein method [1].

References

1. Borenstein E, Kupiec M, Feldman MW, Ruppin E: **Large-scale reconstruction and phylogenetic analysis of metabolic environments.** *Proceedings of the National Academy of Sciences of the United States of America / PNAS* 2008, **105**(38):14482–14487.
