# Supplementary material for: Exploration of the core metabolism of symbiotic bacteria
Source: BMC Genomics. 2012 Aug 31;13:438. doi: 10.1186/1471-2164-13-438 (PMC3543179; doi:10.1186/1471-2164-13-438)
Supplement: Additional file 12 — Potential inputs common to groups of lifestyle. Additional file 12: Table S7: size of the mean, union and intersections of the potential input sets among the different lifestyle groups. [file 1471-2164-13-438-S12.pdf]

Table S7: **Potential inputs common to groups of lifestyle**

	Potential Inputs			Potential Inputs + distance one			
	Mean	Union	Intersection	Mean	Union	Intersection	Intersection
MIV	50	237	0	75	307	0	
PIV	66	138	12	92	183	22	
PIH	64	113	16	98	161	34	
Intra	55	317	0	81	385	0	
MCAV	96	167	26	140	231	50	
PCAH	138	747	0	198	865	1	acetate
CA	133	775	0	191	875	1	acetate
Intra+CA	95	873	0	138	930	0	
MEH	235	433	78	324	556	130	
CEH	180	688	4	253	823	7	
PEH	184	692	5	254	815	17	
Extra	190	944	2	265	1044	2	peptidylproline ($\omega = 180$) and ($\omega = 0$)
FL	164	554	9	234	688	19	
Extra+FL	184	1018	2	257	1105	2	peptidylproline ($\omega = 180$) and ($\omega = 0$)
Total	133	1191	0	189	1191	0	

Size of the mean, union and intersections of the potential input sets among the different lifestyle groups.
